# Cell Cycle Regulated Phosphorylation of the Telomere-Associated Protein TIN2

**DOI:** 10.1371/journal.pone.0071697

**Published:** 2013-08-16

**Authors:** Shuqun Yang, Christopher M. Counter

**Affiliations:** Department of Pharmacology and Cancer Biology, Department of Radiation Oncology, DUMC, Durham, North Carolina, United States of America; University of North Carolina, United States of America

## Abstract

The protein TIN2 is a member of telomere-binding protein complex that serves to cap and protect mammalian chromosome ends. As a number of proteins in this complex are phosphorylated in a cell cycle-dependent manner, we investigated whether TIN2 is modified by phosphorylation as well. We performed phospho-proteomic analysis of human TIN2, and identified two phosphorylated residues, serines 295 and 330. We demonstrated that both these sites were phosphorylated during mitosis in human cells, as detected by Phos-tag reagent and phosphorylation-specific antibodies. Phosphorylation of serines 295 and 330 appeared to be mediated, at least in part, by the mitotic kinase RSK2. Specifically, phosphorylation of TIN2 at both these residues was increased upon expression of RSK2 and reduced by an inhibitor of the RSK family of kinases. Moreover, RSK2 phosphorylated TIN2 *in vitro*. The identification of these specifically timed post-translational events during the cell cycle suggests a potential mitotic regulation of TIN2 by phosphorylation.

## Introduction

The telomere-associated protein 1 interacting nuclear factor 2 (TIN2, TINF2, DKCA3) is a 40kDa protein integral to telomere function. TIN2 has no known enzymatic activity, but interacts with double-stranded (ds) and single-stranded (ss) telomeric DNA-binding proteins [1], [2]. Specifically, TIN2 directly binds to the telomeric dsDNA-binding proteins TRF1 and TRF2, and to TPP1, which forms a heterodimer with the telomeric ssDNA-binding protein POT1 [1], [3]–[5]. Knockdown of TIN2 in human cells results in moderate telomere elongation, as well as destabilization of TRF1 and TRF2 [5], [6]. Knockout of TIN2 in murine embryonic fibroblasts results in severe telomere dysfunction phenotypes such as high levels of telomere-dysfunction induced foci, telomere fusions, as well as telomere sister chromatid exchange (T-SCE) [7].

Circumstantial evidence suggests that TIN2 might be phosphorylated in a cell cycle dependent fashion. First, the increase in T-SCE upon loss of TIN2 suggests a critical role for TIN2 in mitosis [7], and many proteins are regulated at mitosis by phosphorylation [8]. Second, TIN2 is found in a complex with TRF1 and cohesins [9], the latter of which are functionally regulated by phosphorylation during mitosis [10]–[12]. Third, at least two other telomere proteins have been reported to be phosphorylated. Specifically, TRF1 is phosphorylated at mitosis to allow sister telomere resolution [13], and the phosphorylation of TPP1 during G2/M phase is related to higher telomerase activity [14]. Given these observations, we explored the possibility that TIN2 may be phosphorylated, perhaps at mitosis.

## Results

### Mass Spectrometry Identifies Two Phosphorylated Residues in TIN2

**Figure 1:**
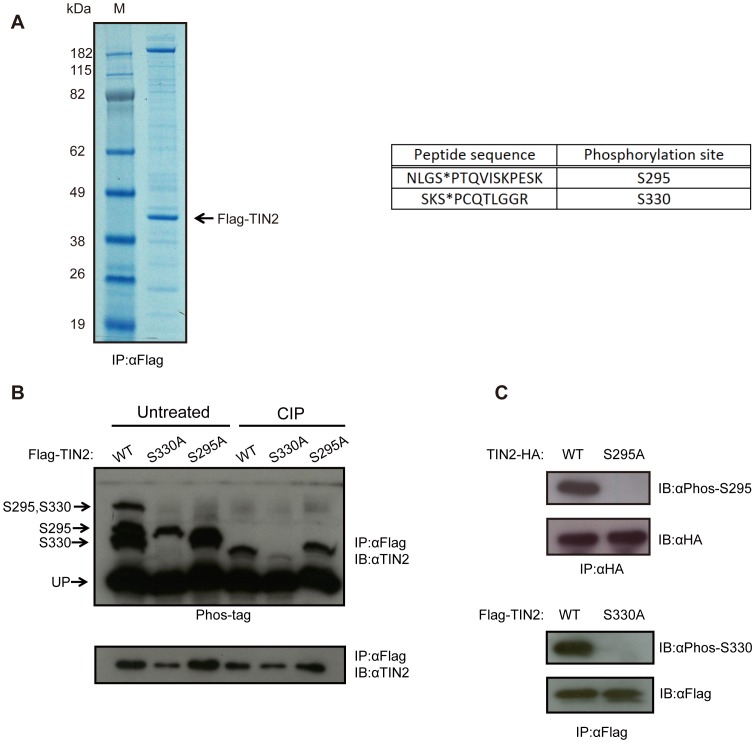
TIN2 is phosphorylated on Serine 295 and Serine 330. (**A**) Identification of phosphorylation sites on TIN2 by mass spectrometry. *Left,* A lysate from HeLa cells stably infected with a retrovirus encoding N-terminal Flag epitope-tagged TIN2 (Flag-TIN2) was subjected to immunoprecipitation (IP) with an anti-Flag antibody, resolved by SDS-PAGE, and detected by Coomassie Brilliant Blue staining. M: marker lane. *Right,* The purified protein was then recovered and digested by trypsin, followed by TiO2 enrichment and mass spectrometry analysis, revealing two peptides with phosphorylated serine residues (denoted with an *). Representative of one experiment. (**B**) Detection of phosphorylation of TIN2 at S295 and S330 by the Phos-tag reagent. Lysates from HeLa cells stably infected with a retrovirus encoding Flag-TIN2 in the wild-type (WT), S330A, or S295A configuration were subjected to immunoprecipitation (IP) with an anti-Flag antibody and then either left untreated or treated with calf intestine phosphatase (CIP), followed by SDS-PAGE either in the presence (*top*) or the absence (*bottom*) of the Phos-tag reagent and immunoblotted (IB) with an anti-TIN2 antibody. The supershifted bands corresponding to S295, S330, or S295 and S330 phosphorylation, as well as the unphosphorylated TIN2 (UP), are denoted on the left. Representative of three experiments. (**C**) Detection of S295 and S330 phosphorylation of TIN2 with phosphorylation-specific antibodies. Lysates from HeLa cells stably infected with a retrovirus encoding C-terminal HA epitope-tagged TIN2 (TIN2-HA) or Flag-TIN2 in WT, S295A, or S330A configuration were subjected to immunoprecipitation (IP) with either an anti-HA or anti-Flag antibody, resolved by SDS-PAGE, and immunoblotted (IB) with either an anti-Phos-S295 or anti-Phos-S330 antibody to detected the phosphorylated TIN2, and either an anti-HA or anti-Flag antibody to detect total ectopic TIN2 as a loading control. Representative of two experiments.

To investigate whether TIN2 is phosphorylated, we ectopically expressed a Flag epitope-tagged version of the more commonly studied shorter cDNA version of TIN2 (Flag-TIN2) in HeLa cells [15]. This protein was then immunoprecipitated by virtue of the Flag epitope and resolved by SDS-PAGE, revealing primarily one band at 40 kDa, the estimated molecular weight of TIN2 (Figure 1A, *left*). The band was excised and subjected to in-gel tryptic digestion, after which the phospho-peptides were enriched by TiO_2_ affinity chromatography and subjected to mass spectrometry analysis [16]_._ This analysis revealed two phosphorylation sites on TIN2, namely serines (S) 295 and 330 (Figure 1A, *right*). Encouragingly, phosphorylation of TIN2 on S295/T297 and/or S330 were detected in unbiased whole phospho-proteome analysis of HeLa cells throughout the cell cycle [17], HeLa cells treated with the drug rapamycin [18], and in nocodazole-arrested HeLa cells [19]. Given our identification of these two sites, and their detection in unbiased phospho-proteomic screens, we reasoned that S295 and S330 may be *bona fide* phosphorylation sites in TIN2.

### Detection of TIN2 Phosphorylation on S295 and S330 by Phos-tag Analysis

**Figure 2:**
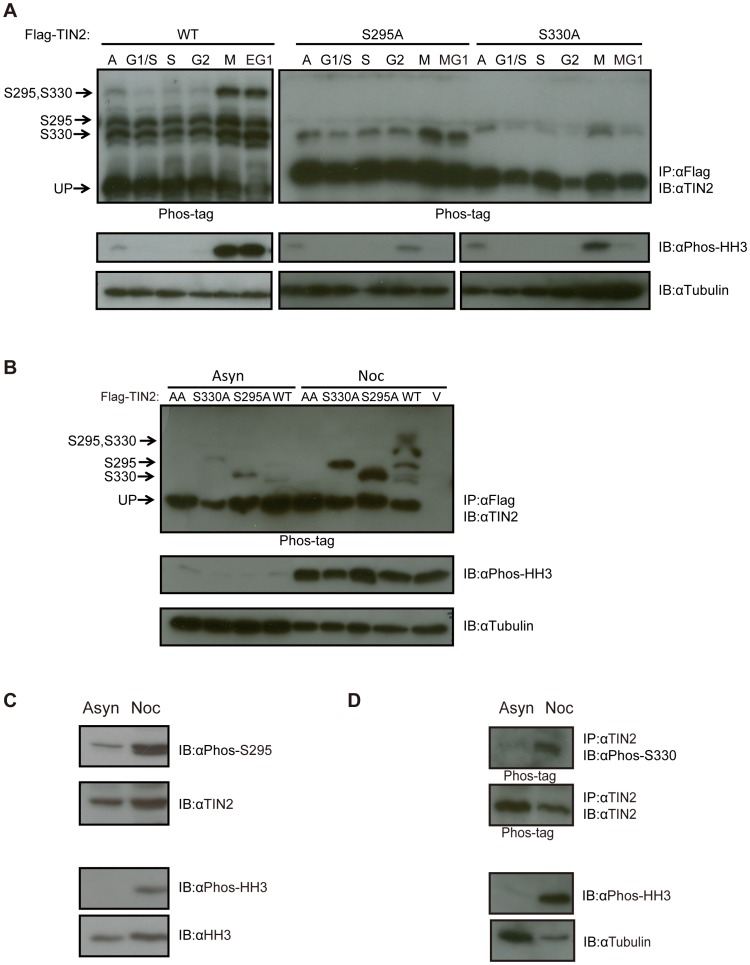
TIN2 is phosphorylated on S295 and S330 during mitosis. (**A**) Detection of S295 and S330 phosphorylation of TIN2 during mitosis by the Phos-tag reagent after release from a double thymidine block. HeLa cells stably infected with a retrovirus encoding Flag-TIN2 in the WT, S330A, or S295A configuration were collected from asynchronous populations (A), populations arrested with a double thymidine block corresponding to the G1/S phase of the cell cycle, or populations at the points corresponding to S, G2, M and early or middle G1 (EG1 or MG1) after release from the double thymidine block. Derived lysates were then either subjected to (*top*) immunoprecipitation (IP) with an anti-Flag antibody and resolved by SDS-PAGE in the presence of the Phos-tag reagent and immunoblotted (IB) with an anti-TIN2 antibody or (*bottom*) resolved by normal SDS-PAGE and immunoblotted with either an anti-Phos-HH3 antibody to monitor cell cycle progression or an anti-Tubulin antibody as a loading control. The supershifted bands corresponding to S295, S330, or S295 and S330 phosphorylation, as well as the unphosphorylated TIN2 (UP) are denoted on the left of the upper panels. Left and right panels are different exposures. Representative of two experiments. (**B**) Detection of S295 and S330 phosphorylation of TIN2 by the Phos-tag reagent in cells arrested with nocodazole. HeLa cells stably infected with a retrovirus encoding no transgene (vector, V) or Flag-TIN2 in the WT, S330A, S295A, or AA configuration were collected from asynchronous populations (Asyn) or populations arrested in G2/M by treatment with nocodazole (Noc). Derived lysates were then subjected to either (*top*) immunoprecipitation (IP) with αFlag and resolved by SDS-PAGE in the presence of the Phos-tag reagent and immunoblotted (IB) with an anti-TIN2 antibody or (*bottom*) resolved by normal SDS-PAGE and immunoblotted with either an anti-Phos-HH3 antibody to monitor cell cycle progression or an anti-Tubulin antibody as a loading control. The supershifted bands corresponding to S295, S330, or S295 and S330 phosphorylation, as well as the unphosphorylated TIN2 (UP), are denoted on the left of the upper panel. Representative of three experiments. (**C**) Detection of S295 phosphorylation of endogenous TIN2 with a phosphorylation-specific antibody in cells arrested with nocodazole. Lysates from HeLa cells were collected from asynchronous populations (Asyn) or populations arrested in G2/M by treatment with nocodazole (Noc), resolved by SDS-PAGE and immunoblotted (IB) with an anti-Phos-S295, anti-TIN2, anti-Phos-HH3, or anti-HH3 (loading control) antibody. Representative of two experiments. (**D**) Detection of S330 phosphorylation of endogenous TIN2 with a phosphorylation-specific antibody in cells arrested with nocodazole. HeLa cells were collected from asynchronous populations (Asyn) or populations arrested in G2/M by treatment with nocodazole (Noc). Derived lysates were then either subjected to (*top*) immunoprecipitation (IP) with an anti-TIN2 antibody, resolved by SDS-PAGE in the presence of the Phos-tag reagent, and immunoblotted (IB) with either an anti-Phos-S330 or anti-TIN2 antibody, or (*bottom*) resolved by normal SDS-PAGE and immunoblotted with an anti-Phos-HH3 antibody, to monitor cell cycle progression, or an anti-Tubulin antibody as a loading control. Representative of one experiment.

**Figure 3:**
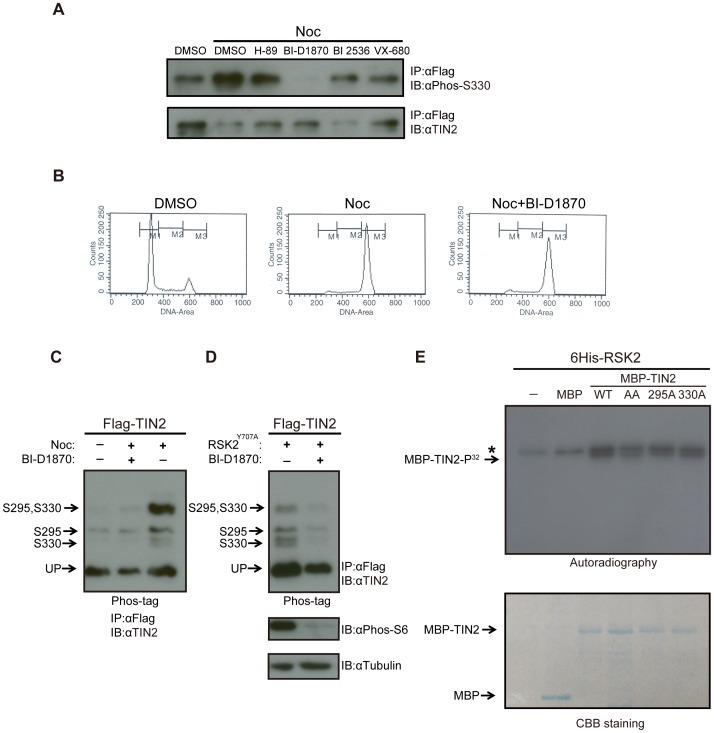
TIN2 is phosphorylated by the mitotic kinase RSK2. (**A**) Detection of S330 phosphorylation of TIN2 with a phosphorylation-specific antibody in cells arrested with nocodazole and treated with kinase inhibitors. HeLa cells stably expressing wild-type Flag-TIN2 were treated with DMSO, H-89, BI-D1870, BI 2536 or VX-680 in the presence of either nocodazole (Noc) or vehicle (DMSO). Derived lysates were immunoprecipitated (IP) with an anti-Flag antibody, resolved by SDS-PAGE, and immunoblotted (IB) with an anti-Phos-S330 antibody or, as a loading control, an anti-TIN2 antibody. Representative of two experiments. (**B**) DNA profiles of HeLa cells treated with BI-D1870. HeLa cells treated with DMSO, nocodazole (Noc), or nocodazole+ BI-D1870 were harvested, stained with propidium iodide, and subjected to fluorescence-activated cell sorting (FACS) analysis. Representative of two experiments. (**C**) Detection of S295 and S330 phosphorylation of TIN2 by the Phos-tag reagent in asynchronous or nocodazole arrested cells with or without the RSK2 inhibitor BI-D1870. 293T cells were either untreated or treated with nocodazole (Noc), BI-D1870, or both compounds. Derived lysates were then subjected to immunoprecipitation (IP) with an anti-Flag antibody and resolved by SDS-PAGE in the presence of the Phos-tag reagent and immunoblotted (IB) with an anti-TIN2 antibody. The supershifted bands corresponding to S295, S330, or S295 and S330 phosphorylation, as well as the unphosphorylated TIN2 (UP), are denoted on the left. Representative of two experiments. (**D**) Detection of S295 and S330 phosphorylation of TIN2 by the Phos-tag reagent in asynchronous cells with ectopic RSK2 and/or the RSK2 inhibitor BI-D1870. 293T cells transiently transfected with Flag-TIN2 and the Y707A constitutively active mutant form of RSK2 (Flag-RSK2^Y707A^) were either left untreated or treated with RSK kinase inhibitor BI-D1870. Derived lysates were split into two portions. The first portions were subjected to immunoprecipitation (IP) with an anti-Flag antibody, resolved by SDS-PAGE in the presence of the Phos-tag reagent, and immunoblotted (IB) with an anti-TIN2 antibody. The supershifted bands corresponding to S295, S330, or S295 and S330 phosphorylation, as well as the unphosphorylated TIN2 (UP), are denoted on the left (*top*). The second portions were resolved by normal SDS-PAGE and immunoblotted with either an anti-Phospho-S6 antibody to monitor RSK2 kinase activity, or an anti-Tubulin antibody as a loading control (*bottom*). Representative of two experiments. (**E**) Detection of TIN2 phosphorylation by RSK2 *in vitro*. Recombinant maltose-binding protein (MBP) or N-terminal MBP-tagged TIN2 (MBP-TIN2) in the WT, S295A, S330A, or AA mutant configuration were captured with amylose resin and eluted with maltose. No protein (-) or equal amounts of the aforementioned purified MBP-TIN2 proteins were incubated with recombinant N-terminal 6His-tagged RSK2 (6His-RSK2) in the presence of ATP^32^, after which the reaction products were resolved by SDS-PAGE and either (*top*) exposed to autographic film or (*bottom*) stained with Coomassie Brilliant Blue (CBB staining). Phosphorylated (P^32^) MBP-TIN2 and a non-specific band (*) are denoted on the left top panel. MBP-TIN2 and MBP are denoted on the left bottom panel. Representative of two experiments.

To determine whether S295 and S330 of TIN2 are indeed phosphorylated, as suggested by mass spectrometry analysis, Flag-TIN2 cDNA was mutated to encode either a S295 to alanine (A) mutation (S295A) or a S330 to A mutation (S330A). These two mutants, as well as a control wild-type version of Flag-TIN2, were stably expressed in HeLa cells. All three proteins were immunoprecipitated by virtue of the Flag tag and resolved by SDS-PAGE containing the dinuclear metal complex Phos-tag reagent, which can specifically bind to phospho groups on proteins and impede their migration [20]. TIN2 was then detected by immunoblot with an anti-TIN2 antibody. This analysis revealed four major bands from lysates derived from HeLa cells expressing wild-type TIN2; one band residing at the molecular weight of TIN2, corresponding to the unphosphorylated protein, and three supershifted bands. The lowest of these supershifted bands was absent in cells stably expressing the S330A TIN2 mutant, indicating that this band corresponds to S330 phosphorylation. Interestingly, this lower supershifted band appeared as either a singlet or doublet (Figures 1B, 2A,B, 3B). As phosphorylation of S2448 of mTOR similarly yields more than one band using the Phos-tag reagent [21], the doublet may represent altered migration of TIN2 when phosphorylated on S330, although other possibilities cannot be excluded. The second supershifted band was absent in cells stably expressing the S295A mutant, indicating that this band corresponds to phosphorylation at S295. The highest supershifted band was absent in cells expressing either of the S295A or S330A TIN2 mutants, indicating that this band corresponds to the doubly phosphorylated protein (Figure 1B, *left*). Finally, the intensity of these supershifted bands were either abolished or greatly reduced by phosphatase treatment, whereas there was no change in the intensity of the band corresponding to the unphosphorylated protein (Figure 1B, *right*), arguing that the three supershifted bands represent the indicated phosphorylation events. As no further bands were detected, and the intensity of all three supershifted bands was diminished by phosphatase treatment, these results support the contention that TIN2 is phosphorylated principally on S295 and S330.

### Detection of TIN2 Phosphorylation on S295 and S330 by Phosphorylation-specific Antibodies

To independently validate the observation that S295 and S330 in TIN2 are phosphorylated, phosphorylation-specific antibodies against these two sites were generated. Specifically, serum was collected from rabbits inoculated with one of the phospho-mimicking peptides PFRNLG[S^P^]PTQVISK or STGKSK[S^P^]PCQTLG. Lysates derived from HeLa cells expressing a C-terminal HA epitope-tagged TIN2 (TIN2-HA) in either the wild-type or S295A mutant configuration were immunoprecipitated with an anti-HA antibody, resolved by SDS-PAGE, and immunoblotted with the antibody against the S295 phosphorylated TIN2 peptide, termed anti-Phos-S295 for ease of description. The antibody detected a band corresponding to the molecular weight of TIN2 in cells expressing wild-type, but not S295A mutant TIN2 (Figure 1C, *top*). Moreover, when ectopic Flag-TIN2 was immunoprecipitated from HeLa cells with an anti-Flag antibody followed by treatment with calf intestinal phosphatase and resolved by SDS-PAGE, the band detected by the anti-Phos-295 antibody was lost (Figure S1A). Similarly, lysates derived from HeLa cells expressing Flag-TIN2 in either the wild-type or S330A mutant configuration were immunoprecipitated with an anti-Flag antibody, resolved by SDS-PAGE, and immunoblotted with the antibody against the S330 phosphorylated TIN2 peptide, termed anti-Phos-S330 for ease of description. This antibody detected a band corresponding to the molecular weight of TIN2 in cells expressing wild-type, but not S330A mutant TIN2 (Figure 2C, *bottom*). Moreover, treatment of the Flag immunoprecipitate with calf intestinal phosphatase reduced the intensity of the Flag-TIN2 band detected by the anti-Phos-330 antibody by roughly half (Figure S1B), although admittedly this was not as large a difference as that seen with the anti-Phos-295 antibody. Thus, the use of phosphorylation-specific antibodies supports the conclusion from the Phos-tag analysis that TIN2 is phosphorylated on S295 and S330.

### TIN2 is Preferentially Phosphorylated on S295 and S330 during Mitosis

As phosphorylation events are often tightly regulated throughout the cell cycle [8] and an unbiased phospho-proteomic analysis revealed that TIN2 was preferentially phosphorylated on S295 in nocodazole-treated HeLa cells [19], we tested whether phosphorylation of TIN2 on S295 and/or S330 varied during the cell cycle. Specifically, HeLa cells stably expressing Flag-TIN2 in the wild-type, S295A, or S330A configurations were arrested at G1/S phase by double thymidine block and then released to progress through cell cycle. Cell cycle phases were assigned based on previous FACS analysis [22] and confirmed by immunoblot detection of serine 10 phosphorylated histone H3, which is elevated during mitosis [23]. No obvious differences in the cell cycle profile, as determined by FACS, were noted among HeLa cells stably infected with a vector encoding no transgene, wild-type Flag-TIN2, or Flag-TIN2 in which both S295 and S330 were mutated to A, termed the AA mutant for ease of description (Figure S2). Flag-TIN2 protein was then immunoprecipitated with an anti-Flag antibody from lysates derived from these three cell lines when untreated (asynchronous), arrested at G1/S by double thymidine block, or at intervals after release from this block that corresponded to S, G2, M, and either early G1 (EG1) or middle G1 (MG1) phases of the cell cycle. Immunoprecipitated Flag-TIN2 was then resolved by Phos-tag integrated SDS-PAGE and detected with an anti-TIN2 antibody. Equal loading was verified by immunoblot detection of tubulin (Figure 2A).

Immunoblot of the Phos-tag integrated SDS-PAGE gel revealed four bands in lysates derived from asynchronous cells expressing wild-type TIN2. Namely those corresponding to unphosphorylated, S295, S330, and to a smaller degree, S295/S330 phosphorylated TIN2. In the immunoprecipitates from extracts of cells enriched in G1/S, S, or G2 phases of the cell cycle, only the bottom two supershifted bands were readily detectable, suggesting that TIN2 is normally unphosphorylated and mono-phosphorylated during these phases of the cell cycle. However, the intensity of these bands increased, and the band corresponding to doubly phosphorylated TIN2 was clearly evident in immunoprecipitates from extracts isolated from cells in M and early G1 (Figure 2A). These results suggest that the level of TIN2 phosphorylation at both S295 and S330 increases at mitosis.

To determine if one site is preferentially phosphorylated at mitosis over the other, we compared the phosphorylation status of TIN2 as above throughout the cell cycle in HeLa cells stably expressing either S295A or S330A mutant Flag-TIN2. As expected, only two bands were detected in asynchronous populations of cells expressing either of these mutant proteins, corresponding to the unphosphorylated protein and TIN2 phosphorylated on S295 or S330. The level of singly phosphorylated TIN2 increased at M and middle G1 phases in HeLa cells expressing either of the mutants, pointing towards a coordinated phosphorylation of TIN2 at mitosis. The S295A mutant variably appeared more strongly phosphorylated, suggesting a possible bias towards phosphorylation of S330 (Figure 2A). In conclusion, TIN2 is phosphorylated on S295 and S330, and phosphorylation of both these sites coordinately increases during mitosis.

### TIN2 Phosphorylation on S295 and S330 in Nocodazole-arrested Cells

To independently validate phosphorylation of TIN2 at mitosis, HeLa cells expressing wild-type, S295A, or S330A Flag-TIN2 were either left untreated (asynchronous) or arrested at mitosis by treatment with nocodazole, as confirmed by elevated levels of phosphorylated histone H3. Flag-TIN2 was then immunoprecipitated by virtue of the Flag-epitope tag followed by resolution using Phos-tag integrated SDS-PAGE and detected by immunoblot with an anti-TIN2 antibody. Again, tubulin served as a loading control (Figure 2B). Consistent with the previous results, cells expressing wild-type TIN2 that were treated with nocodazole exhibited three supershifted bands that were nearly undetectable in the untreated asynchronous population. The increase in the intensity of these bands could be specifically ascribed to phosphorylation of S295 and S330, as nocodazole treatment similarly increased the level of the bands corresponding to S295 and S330 phosphorylated TIN2 in cells expressing the S330A and S295A mutants, respectively. Finally, as an added control, we demonstrate that all three supershifted bands were lost in cells expressing the AA mutant of Flag-TIN2 in which both S295 and S330 were mutated to A (Figure 2B). Thus, TIN2 is phosphorylated on S295 and S330 during mitosis, as assessed by two independent methods of cell synchronization.

### Endogenous TIN2 is Phosphorylated on S295 and S330 at Mitosis

To determine whether endogenous TIN2 is preferentially phosphorylated at mitosis on these two sites, HeLa cells were either left untreated (asynchronous) or treated with nocodazole to arrest cells in mitosis, as validated by the appropriate absence or presence of phosphorylated histone H3, respectively. Lysates from the two cell populations were then immunoblotted with the anti-Phos-S295 antibody, revealing a clear increase in the band corresponding to S295-phosphorylated TIN2 in cells treated with nocodazole (Figure 2C). On the other hand, detecting endogenous S330-phosphorylated TIN2 with the anti-Phos-S330 antibody proved to be more challenging. Specifically, endogenous TIN2 first had to be immunoprecipitated with an anti-TIN2 antibody, followed by detection with the anti-Phos-S330 antibody. Moreover, because of cross-reactivity with unphosphorylated TIN2, which was only manifested at the endogenous level (and not observed with ectopic TIN2, Figure 1C), immunoprecipitated TIN2 needed to be further resolved by Phos-tag integrated SDS-PAGE to separate the phosphorylated species prior to immunoblot with the anti-Phos-S330 antibody. Finally, as both the TIN2 and Phos-S330 antibodies were derived from rabbits, we used a light chain specific secondary antibody, which only detected the doubly phosphorylated species. Nevertheless, this analysis revealed a single band corresponding to S295/S330 phosphorylated TIN2 in HeLa cells treated with nocodazole, but not in asynchronous HeLa cells (Figure 2D). We thus conclude that endogenous TIN2 is similarly phosphorylated on S295 and S330 at mitosis.

### The RSK Inhibitor BI-D1870 Reduces TIN2 Phosphorylation

To identify possible kinases responsible for the mitotic phosphorylation of TIN2, unsynchronized or nocodazole-arrested HeLa cells expressing wild-type Flag-TIN2 were treated with either vehicle alone (DMSO) as a control or small molecular compounds known to inhibit common mitotic kinases. Specifically, cells were treated with 10µm BI-D1870, which is reported to inhibit the family of p90 ribosome S6 kinases (RSK) [24], [25], 10nM BI 2536, which is reported to inhibit Polo-like kinase 1 [26], 10µm VX-680, which is reported to inhibit the family of Aurora kinases [25], [27], and lastly, 10µm H-89, which is reported to inhibit protein kinase A [25]. Derived lysates were then subjected to immunoprecipitation with an anti-Flag antibody followed by immunoblot with either the anti-Phos-S330 antibody to detect phosphorylated TIN2 or an anti-TIN2 antibody to detect total TIN2 protein as a loading control. As expected, S330 phosphorylation was higher in nocodazole-arrested compared to asynchronous HeLa cells. More importantly, S330 phosphorylation was essentially undetectable in cells treated with BI-D1870. The other three inhibitors also reduced the level of phosphorylated TIN2, but much less so compared to BI-D1870 (Figure 3A). Interestingly H-89 and VX-680 are known to inhibit RSK kinases [25], although these results do not exclude the possibility that other mitotic kinases aside from the RSK family may yet phosphorylate TIN2. To rule out the possibility that this loss of TIN2 phosphorylation arose because BI-D1870 permitted cells to exit mitosis, DMSO, nocodazole, and nocodazole+BI-D1870 treated HeLa cells were stained with propidium iodide and the DNA content determined by FACS analysis. Nocodazole treatment increased the number of cells arresting with a 4N content compared to DMSO-treated cells, and this did not change when cells were co-treated with nocodazole and BI-D1870 (Figure 3B). Taken together, these data suggest that the RSK family of kinases may phosphorylate TIN2 during mitosis.

### Cellular TIN2 is Phosphorylated by the Mitotic Kinase RSK2

To evaluate whether RSK kinases can phosphorylate TIN2, 293T cells were engineered to transiently express Flag-TIN2 in the absence or presence of RSK2^Y707A^, a constitutively active version [28] of the RSK2 member of the RSK family of kinases, comprised of RSK1, RSK2, RSK3, and RSK4. Transfected cells were then untreated, treated with nocodazole to arrest cells in mitosis, treated with BI-D1870 to inhibit RSK kinases, or treated with both compounds. Thereafter, Flag-TIN2 was immunoprecipitated with an anti-Flag antibody, resolved by Phos-tag SDS-PAGE, and detected with an anti-TIN2 antibody. As expected, treatment with nocodazole increased all three phosphorylated forms of TIN2 compared to untreated cells, and this increase in mitotic phosphorylation was inhibited by BI-D1870 (Figure 3C). Three supershifted bands were also detected in asynchronous 293T cells expressing RSK2^Y707A^, and the intensity of these bands similarly decreased when the cells were treated with BI-D1870 (Figure 3D). As a control, lysates derived from 293T cells expressing RSK2^Y707A^ were also resolved by SDS-PAGE and immunoblotted with an antibody detecting S235/S236 phosphorylated S6 ribosomal protein, a known substrate of RSK2 [29], or tubulin as a loading control. Consistent with BI-D1870 inhibiting RSK kinases, the level of phosphorylated S6 ribosomal protein was decreased in the cells treated with this compound (Figure 3D). These results suggest that TIN2 is phosphorylated on both S295 and S330 by RSK2 during mitosis.

### TIN2 is Phosphorylated by RSK2 in vitro

To assess whether RSK2 can directly phosphorylate TIN2, a recombinant 6-His epitope-tagged version of wild-type RSK2 was incubated with recombinant and purified maltose-binding protein (MBP)-tagged wild-type, S295A, S330A, and AA mutant TIN2 protein in the presence of P^32^-labeled ATP. Reaction products were resolved by SDS-PAGE and exposed to auto-radiographic film to visualize phosphorylated protein. Equal amounts of recombinant MBP or MBP-TIN2 protein in each reaction were confirmed by Coomassie Brilliant Blue staining. Consistent with previous reports [28], [30], 6His-RSK2 exhibited some auto-phosphorylation. However, recombinant MBP-TIN2 was phosphorylated in the presence of 6His-RSK2, and this phosphorylation was reduced if S295, S330, or especially if both S295 and S330 were mutated (Figure 3E). Further, the phosphorylation level of MBP-TIN2 was increased when incubated with 6His-RSK2, and this increase was reduced with BI-D1870 (Figure S3). Admittedly, not all phosphorylation was lost when both S295 and S330 were mutated. However, since BI-D1870 was quite effective at reducing TIN2 phosphorylation (Figure 3A, 3C, 3D), no other phosphorylated residues were identified in TIN2 by mass spectrometry (Figure 1A), and mutating both S295 and S330 blocked all detectable phosphorylation of TIN2 in cells (Figure 2B), we suspect that this residual phosphorylation may be spurious, although other possibilities cannot be discounted. In summary, these data suggest that RSK2 can phosphorylate TIN2.

## Discussion

We identified two, and only two phosphorylation sites on TIN2 by mass spectrometry analysis, namely S295 and S330. Mutational analysis of these two residues, coupled with two independent phosphorylation detection assays, namely Phos-Tag separation of phosphorylated species and immunoblot with phosphorylation-specific antibodies, confirmed that TIN2 was indeed phosphorylated on both these sites. The phosphorylation was also detected on endogenous TIN2. Furthermore, both sites were preferentially phosphorylated in mitosis, either when cells entered mitosis after release from a double thymidine block or when cells were arrested with nocodazole. Lastly, the mitotic kinase RSK2 was found to phosphorylate TIN2 on S295 and S330 both *in vitro* and *in vivo*. Thus, we identify phosphorylation of S295 and S330 as a new regulated post-translational modification of TIN2.

The consequence of TIN2 phosphorylation during mitosis remains to be determined. Preliminary analysis has failed to uncover any overt difference between wild-type and AA mutant TIN2. Specifically, both wild-type and AA Flag-TIN2 co-immunoprecipitated with the known TIN2-interacting proteins TRF1 and TPP1 (Figure S4). Although TIN2 can be ubiquitinated [31], the levels of wild-type and AA Flag-TIN2 protein after cells were treated with cycloheximide were nevertheless similar (Figure S5). Even though TIN2 can localize to mitochondria [32], the wild-type as well as the S295A, S330A and AA mutant versions of GFP-tagged TIN2 exhibited similar co-localization with the mitochondrial marker Mito-Tracker Red (Figure S6). Finally, although telomere-induced foci (TIF) are observed in *TIN2^−/−^* murine cells [7], both the wild-type and phosphorylation mutants of TIN2 suppressed the number of TIFs induced in HeLa cells by TIN2 shRNA (Figure S7). However, as telomere sister chromatid exchanges are elevated in *TIN2^−/−^* murine cells [7], perhaps phosphorylation is related to this aspect of TIN2 function. Alternatively, S295 and S330 reside close to mutation sites found in dyskeratosis congenital patients [33] that affect binding to heterochromatin protein 1γ and telomere length [34], thus perhaps mitotic phosphorylation of TIN2 is instead involved in telomere length regulation. Finally, as RSK2 phosphorylated TIN2, and inhibiting this kinase in mitotic cells reduced TIN2 phosphorylation, TIN2 phosphorylation may be linked with functions of RSK2. In this regard, RSK2 promotes G2/M transition [35] and maintains spindle assembly checkpoint [36]. In summary, we demonstrate that first, only the two sites S295 and S330 in TIN2 are found to be phosphorylated, second, these two sites are preferentially phosphorylated at mitosis and third, RSK2 can phosphorylate TIN2 on these two residues.

## Materials and Methods

### Plasmids

pBabe-puro-Flag-TIN2^WT^, pBabe-puro-TIN2^WT^-HA, and pEGFP-N1-TIN2^WT^ were generated by introducing, in frame, an N-terminal Flag or a C-terminal HA epitope-tag in the human TIN2 cDNA [22] by PCR and subcloning the resultant cDNA into the EcoRI/HindIII sites of pBabe-puro [37]. pBabe-puro-Flag, pMAL-c2x-Flag and pEGFP-N1 TIN2^S295A^, TIN2^S330A^, and the compound S295A/330A TIN2^AA^ mutant were generated by introducing S295A, S330A, or S295A/S330A mutations into the aforementioned Flag-TIN2^WT^ cDNA and subcloning the resultant cDNAs into the EcoRI/HindIII sites of the pBabe-puro vector, the pMAL-c2x vector (New England Lab), and the XhoI/HindIII sites of the pEGFP-N1 vector (Clontech). pBabe-puro-TIN2^S295A^-HA was generated by introducing the S295A into the aforementioned TIN2^WT^-HA cDNA and subcloning the resultant cDNA into the EcoRI/HindIII sites of the pBabe-puro vector. pQCXIP-Flag-TIN2^WT^ was generated by subcloning the aforementioned Flag-TIN2^WT^ cDNA into the NotI/AgeI sites of the pQCXIP vector (catalogue # 6315, Clontech). pcDNA-Flag-RSK2^Y707A^ was a kind gift from Dr. Sally Kornbluth. pCMV-myc-TRF1 [22] and pEYFP-C1-TPP1 [28] were previously described. pSuper-retro-GFP-Neo-shTIN2-1 and -2 were generated by insert small hairpin RNA against TIN2 (5′-GGAGCACAUUCUUUGCCUG-3′ [38] and 5′- CCAACCCAGGUCAUAUCUAAG-3′) into the BglII/HindIII sites of the pSuper-retro-GFP-Neo vector. All manipulated cDNAs were confirmed correct by sequencing.

### Retroviral Infection

For phospho-proteomic analysis of TIN2, 10^5^ HeLa cells (catalogue # CCL-2, American Type Culture Collection) were stably infected with an amphotrophic retrovirus derived from pQCXIP-Flag-TIN2 and selected for resistance to puromycin, exactly as previously described [39]. For analysis of TIN2 mutants, 10^5^ HeLa cells were infected with amphotrophic retroviruses derived from pBabe-puro encoding no transgene (vector) or Flag-TIN2^WT^, Flag-TIN2^S295A^, Flag-TIN2^S330A^, Flag-TIN2^AA^, TIN2^WT^-HA, or TIN2^S295A^-HA and selected for stable resistance to puromycin, as described above.

### Transient Transfection

For analysis of RSK phosphorylation of TIN2, 10^6^ 293T cells (catalogue # CRL-11268, ATCC) were transiently transfected with pBabe-puro-Flag-TIN2^WT^ and/or pcDNA-Flag-RSK2^Y707A^ using the Fugene reagent (catalogue # E2691, Promega) according to the manufacture’s protocol.

### Protein Purification and Mass Spectrometry

10^8^ HeLa cells stably infected with pBabe-puro-Flag-TIN2^WT^ were lysed with one volume of high salt buffer (20 mM HEPES pH 8.0, 420 mM NaCl, 25% glycerol, 0.1 mM EDTA, 5 mM MgCl_2_ and 0.2% NP-40) supplemented with a protease inhibitor cocktail (catalogue # 11836170001, Roche) and phosphatase inhibitor cocktails (catalogue # P5726 and P0044, Sigma). Cell lysate was diluted with three volume of dilution buffer (20 mM HEPES pH 8.0, 60 mM NaCl, 0.1 mM EDTA, 5 mM MgCl_2_, and 0.07% NP-40) and incubated with M-2 resin (catalogue # A2220, Sigma) overnight at 4°C, then washed three times with lysis buffer at 4°C. Proteins were recovered by IgG elution buffer (catalogue #21004, Thermo Scientific) and resolved by SDS-PAGE on Novex® 4–20% Tris-Glycine Mini Gels. The band corresponding to the molecular weight of TIN2 (40 kDa), identified after Coomassie Brilliant Blue staining, was excised for mass spectrometry analysis by Duke Proteomics Core Facility. In brief, the protein was subjected to in-gel tryptic digestion. Phospho-peptides were then enriched by TiO_2_ affinity chromatography and subjected to mass spectrometry analysis [16].

### Immunoblot Analysis

10^6^ of the described transiently transfected 293T cells or stably infected HeLa cells were lysed with RIPA lysis buffer (50 mM Tris-HCl pH 8.0, 150 mM NaCl, 1% NP-40, 0.1% SDS, 0.5% NaDoc) supplemented with 1 mM PMSF, 1 mM DTT, 1 µg/ml leupeptin, 1 µg/ml aprotinin, 1 µg/ml pepstatin, 1 mM Na_3_VO_4_, and 1 mM NaF. Cell lysate were then centrifuged at 4°C for 10 minutes. Protein concentrations of the supernatants were determined by Bradford assay. Equal amount of protein were mixed with 4x loading buffer (200 mM Tris-HCl pH 6.8, 400 mM DTT, 8% SDS, 0.4% bromophenol blue, and 40% glycerol) and boiled for 5 minutes. SDS-PAGE gels were made either with or without 30 µM Phos-tag and 60 µM MnCl_2_, according to manufacturer’s protocol (catalogue # Phos-tag AAL-107, Wako Pure Chemical Industries). After electrophoresis, proteins were transferred onto PVDF membranes (catalogue # IPVH00010, EMD Millipore) at 4°C. The membranes were blocked with 5% non-fat milk in TBST, then incubated with first antibody at 4°C overnight. The membranes were washed twice with TBST (0.05 M Tris pH 7.4, 0.15 M NaCl, and 0.02% Tween-20) at room temperature and then incubated with secondary antibody for one hour at room temperature. Finally, the membranes were incubated with ECL (catalogue # RPN2106, General Electric Healthcare) and exposed to hyper-film (catalogue # 28-9068-39, GE Healthcare). In some cases, the membranes were stripped with 7 M GuCl and 10 mM DTT, extensively washed in ddH_2_O at room temperature and blocked again with 5% non-fat milk in TBST. Stripped membranes were then re-probed with another antibody. The following antibodies and concentrations were used: mouse anti-beta-tubulin at 1∶1000 (catalogue # T5201, Sigma), mouse anti-Flag at 1∶1000 (catalogue # F1804, Sigma), mouse anti-HA at 1∶1000 (catalogue # 2367, Cell Signaling Technology), rabbit anti-phospho-histone H3-Ser10 at 1∶1000 (catalogue # 9701, CST), rabbit anti-Phospho-S6 Ribosomal Protein (Ser235/236) at 1∶1000 (catalogue # 2211, CST), rabbit anti-TIN2 [3] at 1∶1000 (a kind gift from Dr. Susan Smith), rabbit anti-phospho-TIN2 Ser295 and rabbit anti-phospho-TIN2 Ser330 at 1∶500 (Pierce customized antibodies derived from peptides PFRNLG[S^P^]PTQVISK and STGKSK[S^P^]PCQTLG, respectively).

### Immunoprecipitation and Phosphatase Treatment

10^6^ of the described transiently transfected 293T cells or stably infected HeLa cells were lysed with RIPA lysis buffer as above. Equal amount of cell lysate were mixed with pre-washed Gamma beads (catalogue # 17-0885-01, GE Healthcare). The mixtures were rotated at 4°C for two hours. 4 µg anti-Flag, anti-HA, or anti-TIN2 antibodies were added and incubated at 4°C on an end-to-end rotator overnight. The beads were then isolated by centrifuge and washed three times with RIPA lysis buffer at 4°C. Beads were boiled with 4x loading buffer for 5 minutes, and supernatants resolved by SDS-PAGE. In the case of phosphatase treatment, lysates were prepared in the absence of phosphatase inhibitors and subjected to immunoprecipitation as above. The protein-bound resins were washed as above and then either left untreated or treated with calf intestinal alkaline phosphatase (catalogue # M0290S, New England BioLabs) for one hour at room temperature. The resins were boiled with 4x loading buffer for 5 minutes and the supernatants resolved by SDS-PAGE in the presence of Phos-tag reagent, as above.

### Cell Synchronization by Nocodazole Arrest and Double Thymidine Block

10^6^ of the described stably infected HeLa cells were either left untreated, arrested in G2/M by overnight treatment with 0.6 µg/ml nocodazole (catalogue # M1404, Sigma), or synchronized at G1/S by the double thymidine block, as outlined below. In some cases, HeLa or 293T cell lines were treated with 10 µM H-89 (Sigma), 10 µM BI-D1870 (Enzo Life Science), 10 nM BI 2536 (Selleckbio), or 1 µM VX-680 (Selleckbio) for 30 minutes in the presence of nocodazole before harvesting. For double thymidine block, HeLa cells were treated with 2 mM thymidine (catalogue # T9250, Sigma) for 19 hours and washed three times with PBS. Cells were then incubated fresh medium without addition of thymidine for 9 hours and treated with thymidine for another 16 hours. Cells were again washed with PBS three times, released into normal fresh medium and collected at 0, 4, 8, 10, 12 or 15 hours, denoted as G1/S, S, G2, M, Early G1 (EG1) and Middle G1 (MG1) phase of cell cycle [22]. Untreated cells or cells at various states of cell cycle were collected and lysed for immunoprecipitation.

### Fluorescence-activated Cell Sorting (FACS)

10^6^ HeLa cells were harvested, washed with PBS, and fixed with methanol for 10 minutes at room temperature. Cells were then treated with 2 mg/ml RNase A solution (catalogue # R4642, Sigma) for 30 minutes at 37°C and stained with 0.1 mg/ml propidium iodide (catalogue # P4170, Sigma) for 30 minutes at 37°C. HeLa cells were then washed in PBS and sorted on a FACSCalibur flow cytometer (Becton Dickson) with CELLQUEST software.

### *In vitro* RSK2 Kinase Assay

Maltose-binding protein (MBP) and MBP tagged TIN2 (MBP-TIN2) were purified from BL21 competent bacteria. Specifically, the bacteria were lysed with lysis buffer (50 mM Tris-HCl pH8.0, 150 mM NaCl, 1% Triton X-100, and 10% glycerol) and then immunoprecipitated with amylose resin for two hours at room temperature. The resins were washed with lysis buffer at room temperature for three times, following elution with lysis buffer+10 mM maltose. Purified proteins were dialyzed against 50 mM Tris-HCl pH8.0 and 150 mM NaCl overnight at 4°C. 2 µg of purified MBP, MBP-TIN2^WT^, MBP-TIN2^S295A^, MBP-TIN2^S330A^, and MBP-TIN2^AA^ protein were incubated with 0.2 µg 6His-RSK2 (catalogue # ab60881, Abcam) in kinase assay buffer (25 mM HEPES, pH 7.4, 10 mM MgCl_2_, 1 mM EGTA, 0.2 mM EDTA, 1 mM DTT, 1 mM Na_3_VO_4_, 50 µM cold ATP, and 5 µCi ATP^32^) for 30 minutes at 30°C. The reactions were then mixed with 4x loading buffer, boiled for 5 minutes, and resolved by SDS-PAGE. Acrylamide gels were stained by Coomassie Brilliant Blue and exposed to auto-radiographic film (catalogue # 27601, GE Healthcare).

### Protein Stability Analysis

293T cells transiently transfected with pBabe-puro-flag-TIN2-WT, -295A, -330A, and -AA were treated with 100 µg/ml cycloheximide (catalogue # C4859, Sigma) for 0, 1, 2, 4, 6 and 8 hours. Cells were harvested and lysed with RIPA buffer. Lysates were resolved by SDS-PAGE and immunoblotted with anti-Flag and anti-Tubulin antibodies. Intensity of Flag-TIN2 bands were quantified by ImageJ, normalized to that of tubulin, and plotted against treatment time.

### Immunofluorescence

HeLa cells transiently transfected with pEGFP-N1- TIN2-WT, -295A, -330A and -AA were seeded on coverslips. 24 hours later, cells were stained with MitoTracker Red CMXRos 1mM (catalogue # M-7512, Invitrogen) for 10 min at 37°C and then fixed, permeabilized, blocked, and mounted as previously described [28]. Co-localization of GFP fusion proteins and MitoTracker Red were assessed by Zeiss Axio Imager wide-field fluorescence microscope.

### Telomere-dysfunction Induced Foci (TIF) Assay

HeLa cells stably infected with pBabe-puro-TIN2^WT^ or -TIN2^AA^ were transiently transfected with pSuper-retro-GFP-Neo-shTIN2-1 and -2 and then seeded on coverslips. 24 hours later, cells were fixed, permeabilized, and blocked as previously described [7]. Coverslips were incubated with an anti-γH2AX antibody at 1∶200 dilution for 1 hour at room temperature and then incubated with a donkey anti-mouse antibody conjugated with rhodamine RedX (Jackson Immuno-Research) at 1∶200 dilution for 30 minutes at room temperature. After extensive washes with PBS at room temperature, cells were dehydrate in ethanol and denatured in pre-warmed 70% formamide (Catalogue #F7508, Sigma)/2X saline-sodium citrate (300 mM NaCl and 30 mM trisodium citrate) at 75°C for 10 minutes, followed by hybridization with 120 nM Cy5-conjugated PNA Telomere C probe (Catalogue #F1003-5, PANAGENE) at 4°C overnight. Coverslips were then washed extensively in 10 mM Tris pH 7.5, 70% formamide 70%, and 0.1% BSA at room temperature, extensively washed in 0.08% Tween-20, 0.15 M NaCl and 0.1 M Tris pH 7.5 at room temperature, and then mounted in anti-fade solution (Catalogue #P-36931, Invitrogen). Co-localization of the telomere probe signal and the γH2AX signal in GFP-positive cells were blindly counted by overlaying the Cy5 and red channels. The difference of total TIFs in each genotype was assessed by student t-test.

## Supporting Information

Figure S1 **Immunoblot with TIN2 phosphorylation-specific antibodies after phosphatase treatment.** Lysates from HeLa cells stably infected with a retrovirus encoding Flag-TIN2 were subjected to immunoprecipitation (IP) with an anti-Flag antibody and then either treated with vehicle (DMSO) or calf intestine phosphatase (CIP), followed by SDS-PAGE and immunoblot (IB) with an anti-TIN2 antibody in addition to an (**A**) anti-Phos-S295 or (**B**) anti-Phos-S330 antibody. Bar graphs depict the relative level of the anti-Phos-S295 or anti-Phos-S330 signal normalized to total TIN2. Representative of one experiment.
(TIF)

Figure S2 **FACS analysis of HeLa cells expressing wild-type or AA mutant TIN2.** HeLa cells stably infected with retroviruses encoding no transgene (vector only), Flag-TIN2^WT^, or Flag-TIN2^AA^ were harvested, stained with propidium iodide, and subjected to fluorescence-activated cell sorting (FACS) analysis. Representative of two experiments.
(TIF)

Figure S3 ***In vitro*** **phosphorylation of TIN2 by RSK2.** Recombinant N-terminal MBP-tagged TIN2 (MBP-TIN2) in the absence or presence of recombinant N-terminal 6His-tagged RSK2 (6His-RSK2) and/or BID-1870 were incubated with ATP^32^. Reaction products were resolved by SDS-PAGE and either (*top*) exposed to autographic film or (*bottom*) stained with Coomassie Brilliant Blue (CBB staining). Arrow: Phosphorylated (*top*) or purified (*bottom*) MBP-TIN2. Representative of one experiment.
(TIF)

Figure S4 **Co-immunoprecipitation of wild-type and AA mutant TIN2 with TPP1 and TRF1.** Cell lysates from 293T cells transiently co-transfected with a pBabe-puro vector (V), pBabe-puro-Flag-TIN2^WT^ (WT), or pBabe-puro-Flag-TIN2^AA^ (AA) and either (**A**) pEGFP-TPP1 or (**B**) pCMV-myc-TRF1 were subjected to immunoprecipitation (IP) with an anti-Flag antibody, resolved by SDS-PAGE, and immunoblotted (IB) with an anti-Flag, anti-GFP, or anti-Myc antibody. Representative of two experiments.
(TIF)

Figure S5 **Normalized protein level of wild-type and AA mutant TIN2 after treatment of cells with cycloheximide.** 293T cells transiently transfected with pBabe-puro-Flag-TIN2^WT^ (WT, closed circle) or pBabe-puro-Flag-TIN2^AA^ (AA, open circle) were untreated (0 timepoint) or treated with 100 µg/ml cycloheximide for 1, 2, 4, 6, or 8 hours. Lysates were resolved by SDS-PAGE and immunoblotted (IB) with an anti-Flag or anti-Tubulin antibody. The intensity of Flag-TIN2 bands were quantified by imageJ, normalized to that of tubulin, and plotted against time. Representative of two experiments.
(TIF)

Figure S6 **Co-localization analysis of wild-type and phosphorylation mutant versions of TIN2 with Mito-Tracker Red.** Confocal microscope imaging of HeLa cells transiently transfected with pEGFP-N1-TIN2 in the WT, 295A, 330A, or AA configurations. DNA was visualized by staining with DAPI, ectopic TIN2 proteins were visualized by virtue of GFP, mitochondria were visualized by staining with MitoTracker Red, and co-localization between TIN2 and mitochondria were visualized by overlaying the latter two images. Each panel represents one of six cells imaged.
(TIF)

Figure S7 **Analysis of telomere-dysfunction induced foci in TIN2 knockdown cells rescued with wild-type or phosphorylation mutant versions of TIN2.** HeLa cells stably infected with pBabe-puro encoding no transgene (vector) or Flag-TIN2 in the wild-type (WT) or AA configuration were transiently transfected with pSuper-retro-GFP-Neo with no insert (vector) or TIN2 shRNA sequences 1 and 2 (shRNA1,2) verified by (**A**) immunoblot analysis (IB) to reduce endogenous TIN2 levels (immunoblot with anti-Tubulin antibody serves as a loading control). Cells were then (**B**) hybridized *in situ* with a Cy5-labelled PNA telomere C probe and an anti-γH2AX antibody and the number of co-localization of the two probes representing telomere-dysfunction induced foci (TIF) per cell quantitated.
(TIF)
