# Supplementary figures and images for: Cell Cycle Regulated Phosphorylation of the Telomere-Associated Protein TIN2
Source: PLoS One. 2013 Aug 16;8(8):e71697. doi: 10.1371/journal.pone.0071697 (PMC3745427; doi:10.1371/journal.pone.0071697)

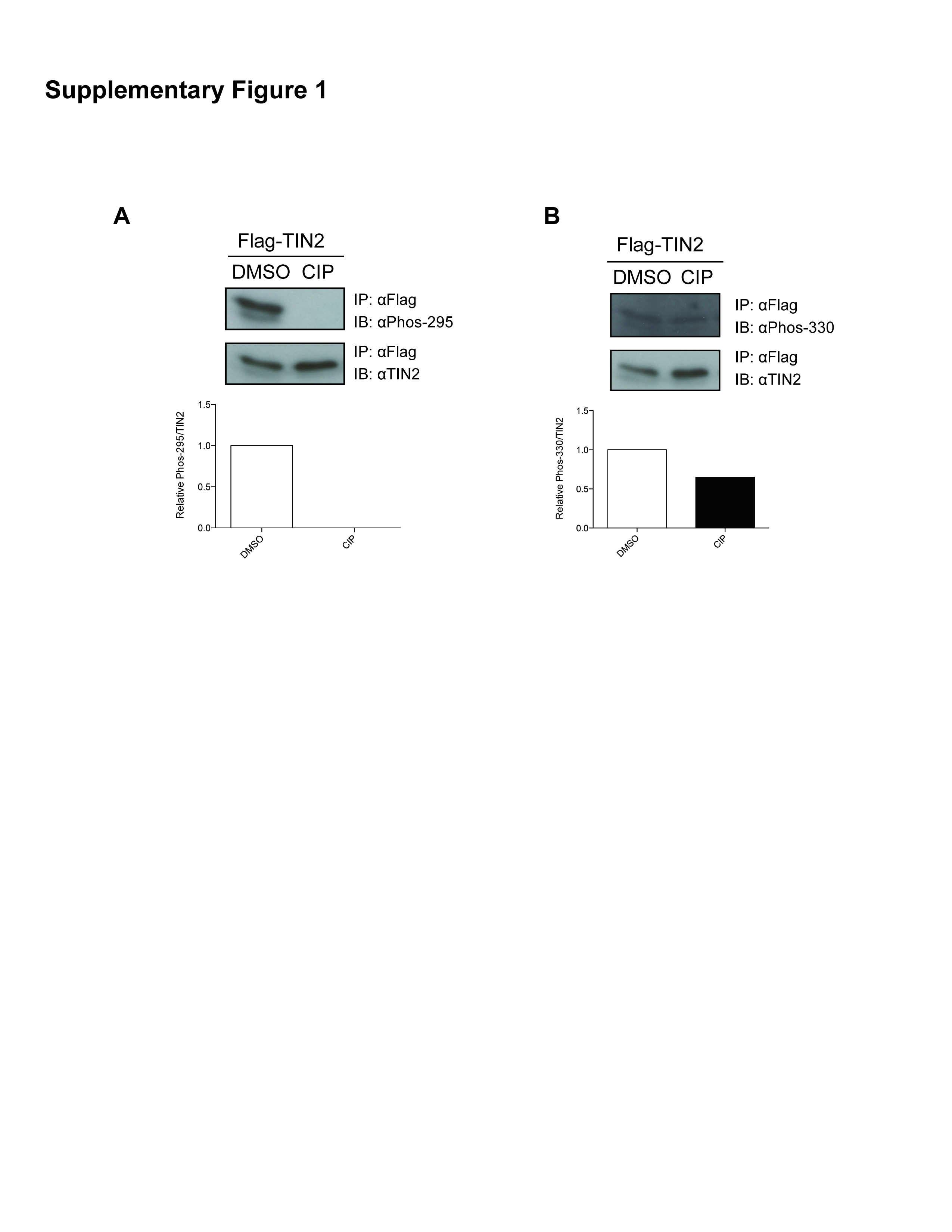

Supplement: Figure S1 — Immunoblot with TIN2 phosphorylation-specific antibodies after phosphatase treatment. Lysates from HeLa cells stably infected with a retrovirus encoding Flag-TIN2 were subjected to immunoprecipitation (IP) with an anti-Flag antibody and then either treated with vehicle (DMSO) or calf intestine phosphatase (CIP), followed by SDS-PAGE and immunoblot (IB) with an anti-TIN2 antibody in addition to an (A) anti-Phos-S295 or (B) anti-Phos-S330 antibody. Bar graphs depict the relative level of the anti-Phos-S295 or anti-Phos-S330 signal normalized to total TIN2. Representative of one experiment. (TIF) [file pone.0071697.s001.tif]

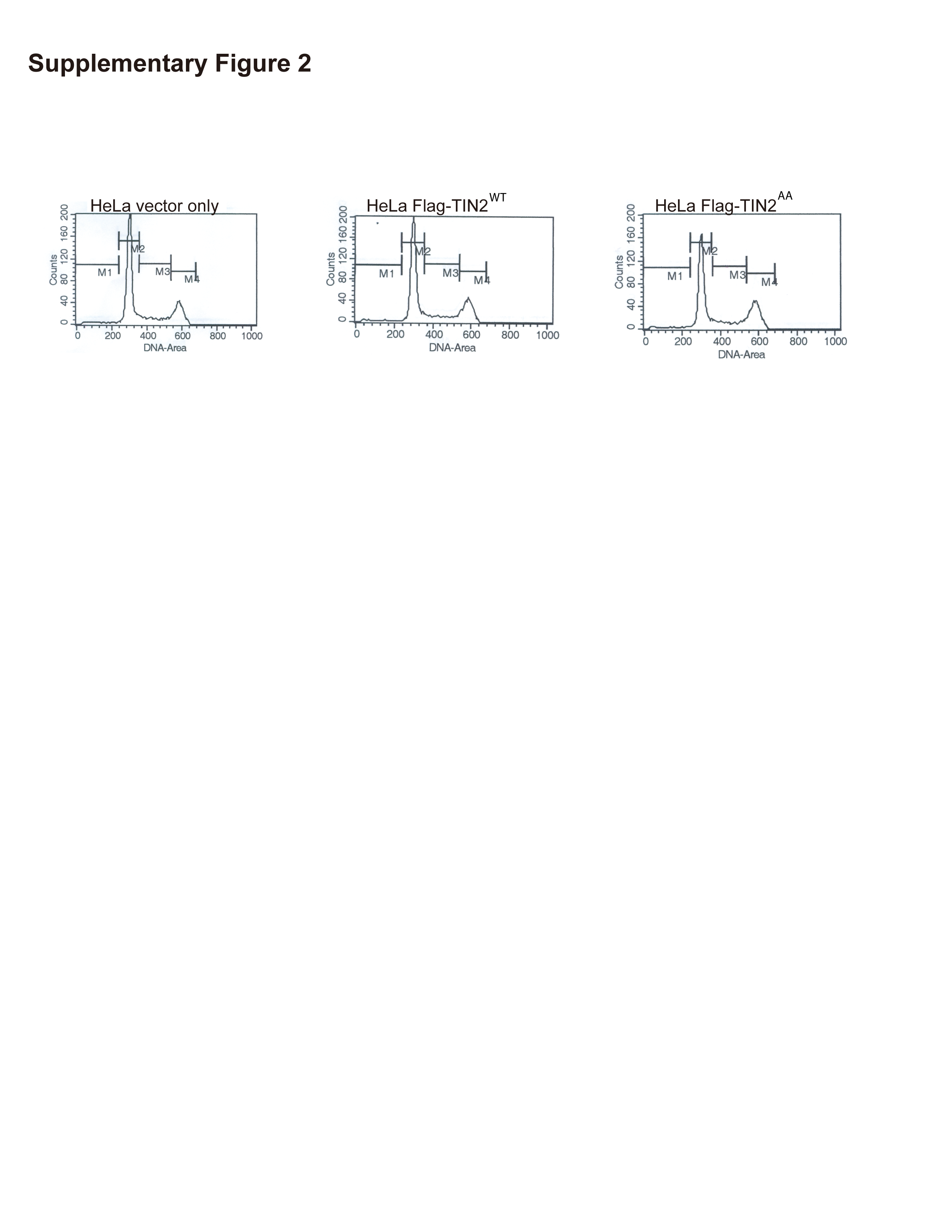

Supplement: Figure S2 — FACS analysis of HeLa cells expressing wild-type or AA mutant TIN2. HeLa cells stably infected with retroviruses encoding no transgene (vector only), Flag-TIN2WT, or Flag-TIN2AA were harvested, stained with propidium iodide, and subjected to fluorescence-activated cell sorting (FACS) analysis. Representative of two experiments. (TIF) [file pone.0071697.s002.tif]

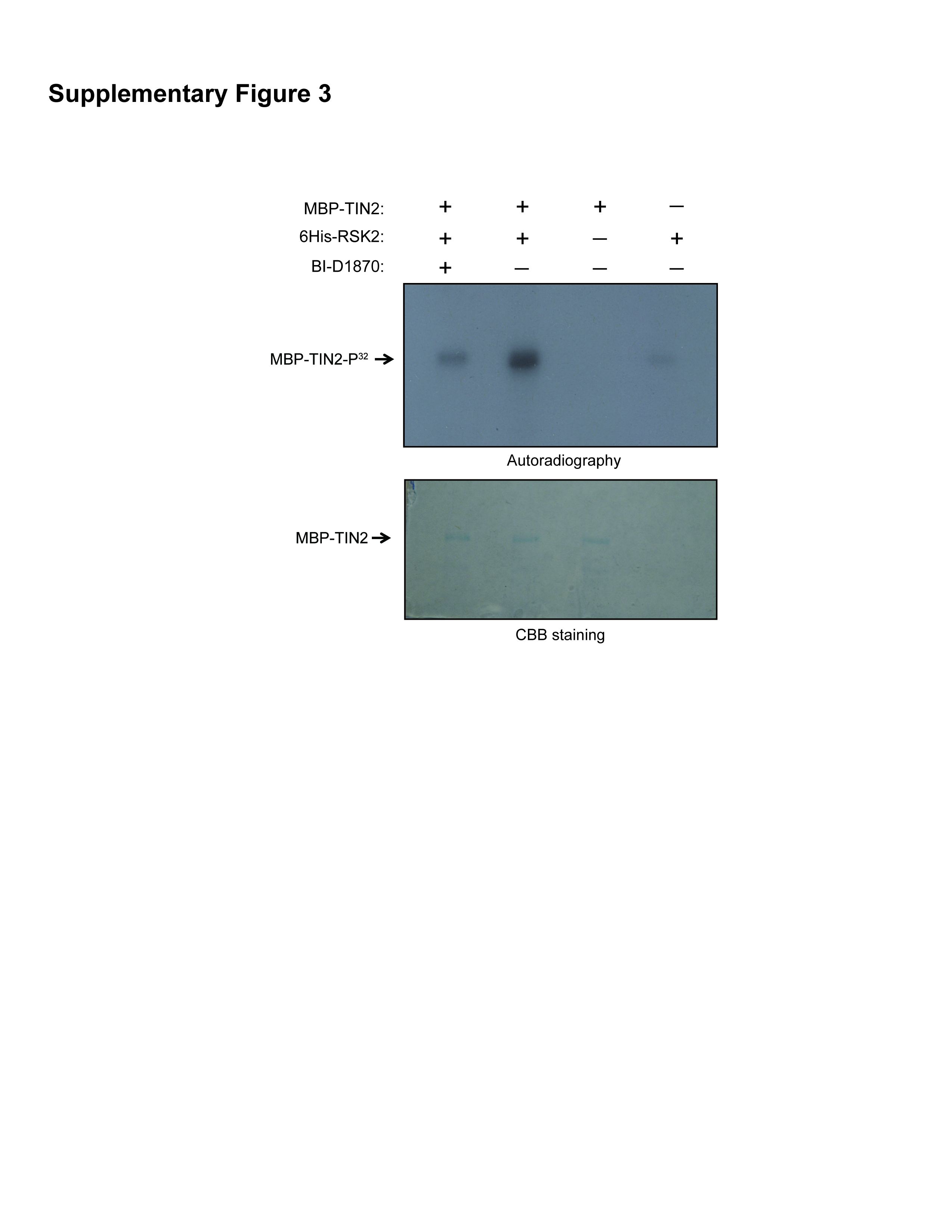

Supplement: Figure S3 — In vitro phosphorylation of TIN2 by RSK2. Recombinant N-terminal MBP-tagged TIN2 (MBP-TIN2) in the absence or presence of recombinant N-terminal 6His-tagged RSK2 (6His-RSK2) and/or BID-1870 were incubated with ATP32. Reaction products were resolved by SDS-PAGE and either (top) exposed to autographic film or (bottom) stained with Coomassie Brilliant Blue (CBB staining). Arrow: Phosphorylated (top) or purified (bottom) MBP-TIN2. Representative of one experiment. (TIF) [file pone.0071697.s003.tif]

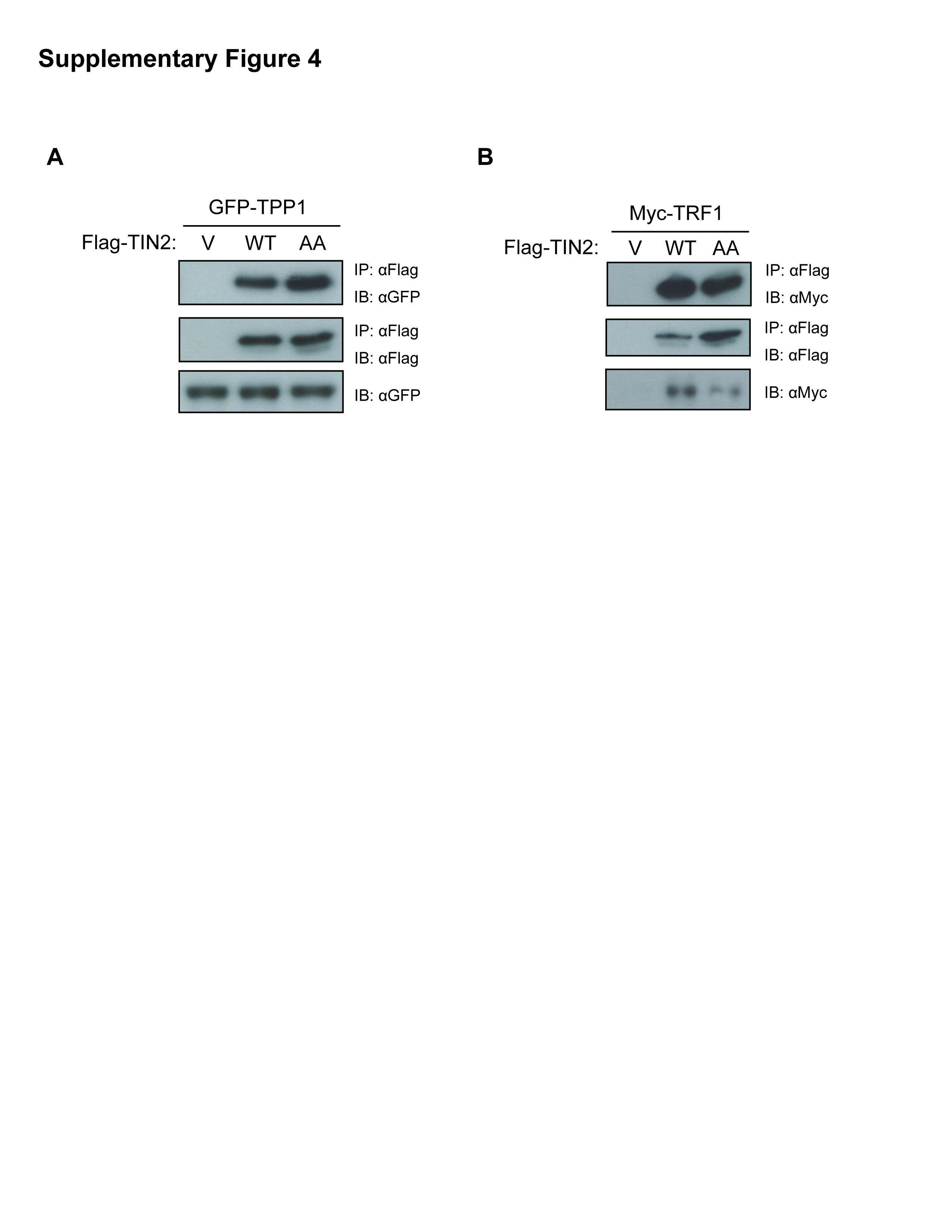

Supplement: Figure S4 — Co-immunoprecipitation of wild-type and AA mutant TIN2 with TPP1 and TRF1. Cell lysates from 293T cells transiently co-transfected with a pBabe-puro vector (V), pBabe-puro-Flag-TIN2WT (WT), or pBabe-puro-Flag-TIN2AA (AA) and either (A) pEGFP-TPP1 or (B) pCMV-myc-TRF1 were subjected to immunoprecipitation (IP) with an anti-Flag antibody, resolved by SDS-PAGE, and immunoblotted (IB) with an anti-Flag, anti-GFP, or anti-Myc antibody. Representative of two experiments. (TIF) [file pone.0071697.s004.tif]

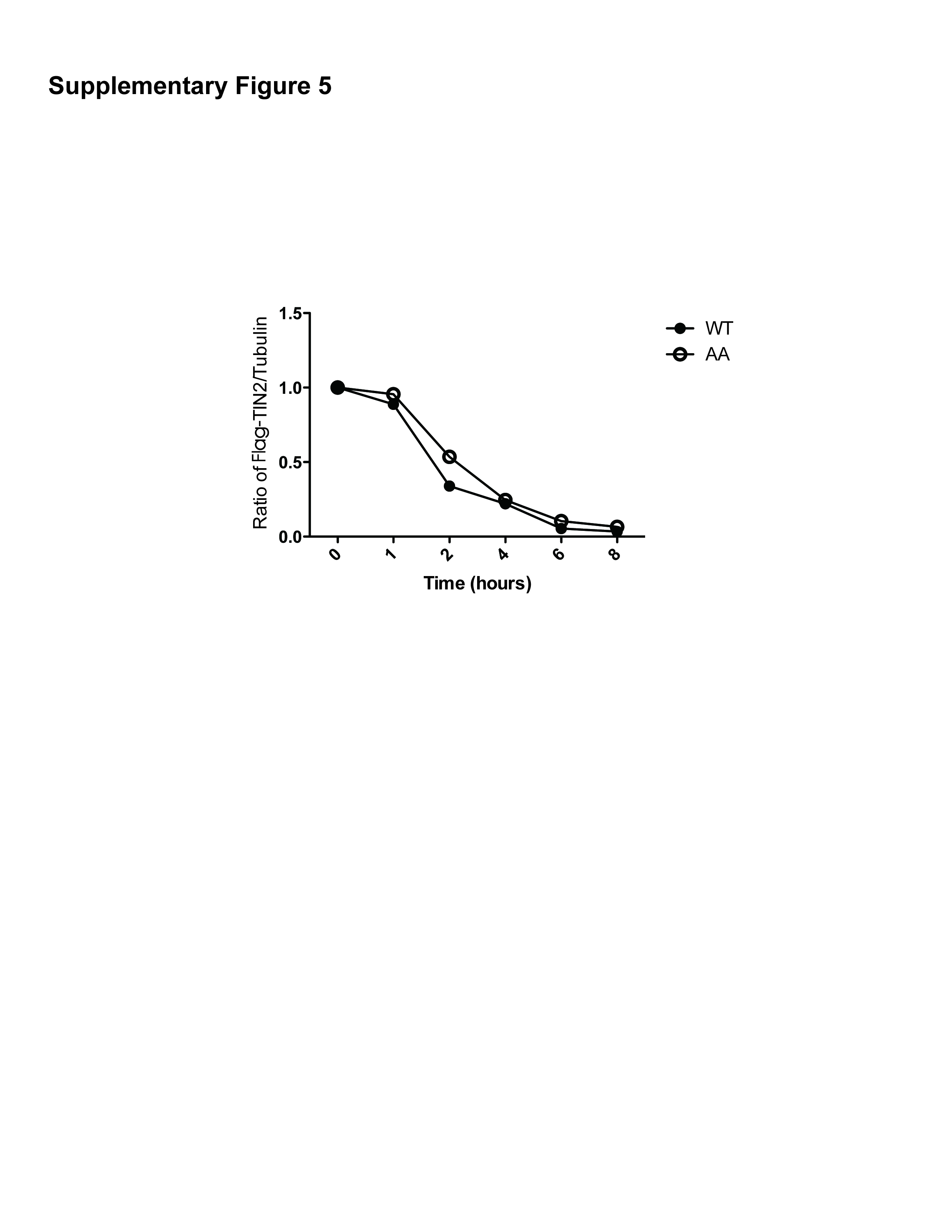

Supplement: Figure S5 — Normalized protein level of wild-type and AA mutant TIN2 after treatment of cells with cycloheximide. 293T cells transiently transfected with pBabe-puro-Flag-TIN2WT (WT, closed circle) or pBabe-puro-Flag-TIN2AA (AA, open circle) were untreated (0 timepoint) or treated with 100 µg/ml cycloheximide for 1, 2, 4, 6, or 8 hours. Lysates were resolved by SDS-PAGE and immunoblotted (IB) with an anti-Flag or anti-Tubulin antibody. The intensity of Flag-TIN2 bands were quantified by imageJ, normalized to that of tubulin, and plotted against time. Representative of two experiments. (TIF) [file pone.0071697.s005.tif]

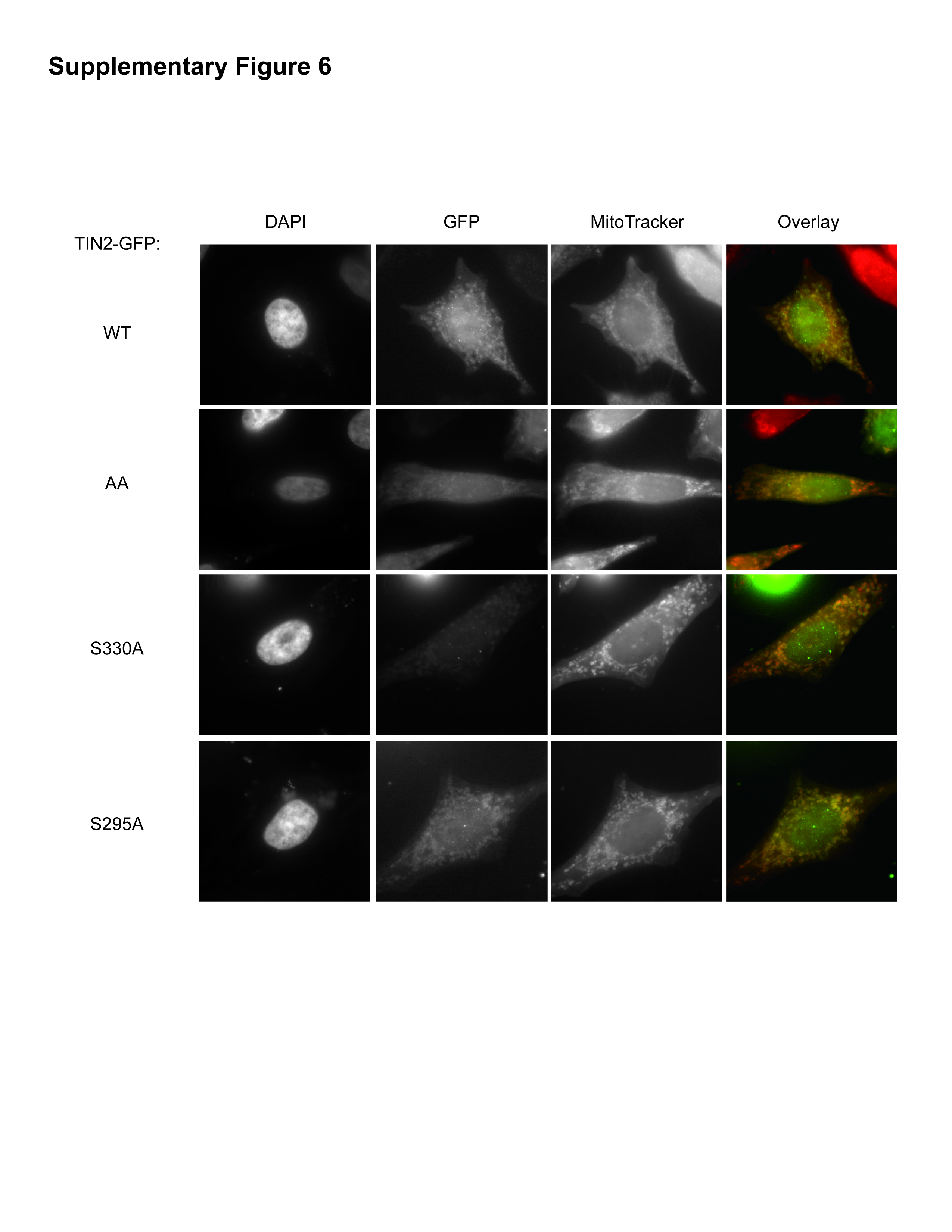

Supplement: Figure S6 — Co-localization analysis of wild-type and phosphorylation mutant versions of TIN2 with Mito-Tracker Red. Confocal microscope imaging of HeLa cells transiently transfected with pEGFP-N1-TIN2 in the WT, 295A, 330A, or AA configurations. DNA was visualized by staining with DAPI, ectopic TIN2 proteins were visualized by virtue of GFP, mitochondria were visualized by staining with MitoTracker Red, and co-localization between TIN2 and mitochondria were visualized by overlaying the latter two images. Each panel represents one of six cells imaged. (TIF) [file pone.0071697.s006.tif]

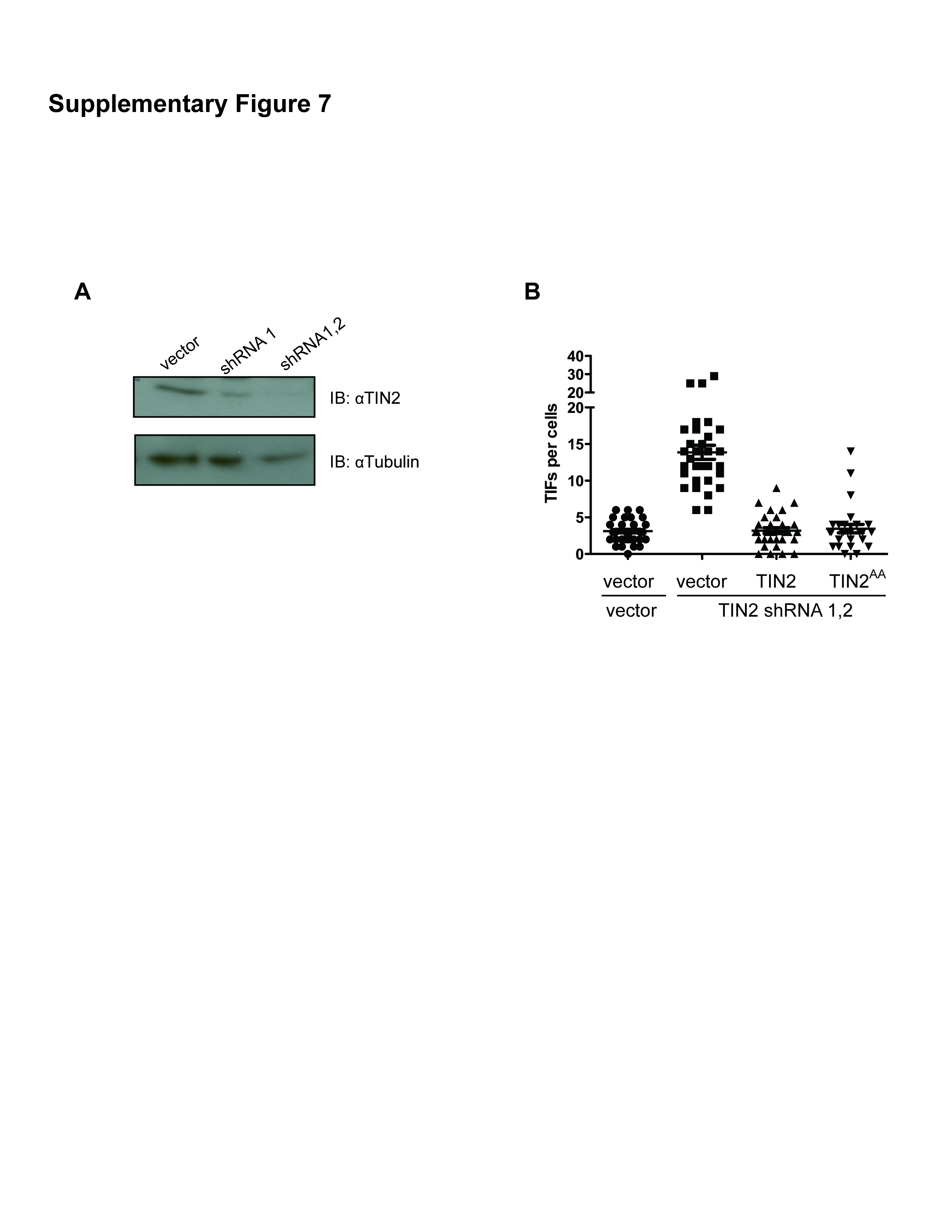

Supplement: Figure S7 — Analysis of telomere-dysfunction induced foci in TIN2 knockdown cells rescued with wild-type or phosphorylation mutant versions of TIN2. HeLa cells stably infected with pBabe-puro encoding no transgene (vector) or Flag-TIN2 in the wild-type (WT) or AA configuration were transiently transfected with pSuper-retro-GFP-Neo with no insert (vector) or TIN2 shRNA sequences 1 and 2 (shRNA1,2) verified by (A) immunoblot analysis (IB) to reduce endogenous TIN2 levels (immunoblot with anti-Tubulin antibody serves as a loading control). Cells were then (B) hybridized in situ with a Cy5-labelled PNA telomere C probe and an anti-γH2AX antibody and the number of co-localization of the two probes representing telomere-dysfunction induced foci (TIF) per cell quantitated. (TIF) [file pone.0071697.s007.tif]
